# Thymidine phosphorylase in nucleotide metabolism: physiological functions and its implications in tumorigenesis and anti-cancer therapy

**DOI:** 10.3389/fimmu.2025.1561560

**Published:** 2025-04-15

**Authors:** Bo Huang, Qihang Yuan, Jiaao Sun, Chao Wang, Dong Yang

**Affiliations:** ^1^ Liaoning Cancer Hospital & Institute, Shenyang, China; ^2^ First Affiliated Hospital of Dalian Medical University, Dalian, China

**Keywords:** nucleotide metabolism, thymidine phosphorylase, physiological functions, tumorigenesis, anticancer therapy

## Abstract

Thymidine phosphorylase (TYMP), a protein found in both prokaryotic and eukaryotic cells, is encoded by a gene located in the q13 region of chromosome 22. With a relative molecular mass of 55,000, TYMP exists as a homodimer. Recent research has increasingly illuminated the diverse functions of TYMP. It is known to facilitate platelet activation, osteoclast differentiation, and angiogenesis. Mutations in the TYMP gene are linked to mitochondrial neurogastrointestinal encephalomyopathy. Beyond its physiological roles, TYMP contributes significantly to tumor growth and cancer progression, where it promotes angiogenesis, modulates epigenetic genes, inhibits apoptosis, and acts as a critical enzyme in the nucleoside metabolic rescue pathway. Moreover, TYMP holds substantial implications in cancer treatment and prognosis. Given its involvement in cancer progression, TYMP inhibitors may prove valuable in inhibiting tumor growth and metastasis. Interestingly, while TYMP can drive tumor growth, certain concentrations of TYMP also enhance the cytotoxic effects of chemotherapy drugs such as 5-fluorouracil (5-FU). Although challenges exist—such as the potential disruption of normal physiological functions when inhibiting TYMP—the protein remains a promising target for cancer treatment. Ongoing research on TYMP could deepen our understanding of human physiology and the pathogenesis of cancer and open new avenues for therapeutic interventions. This article provides a comprehensive review of TYMP’s structure, physiological functions, and its role in tumorigenesis and anti-tumor therapy.

## 1 Introduction

Thymidine phosphorylase (TYMP) was first isolated and purified from animal tissues by Friedkin and Roberts in 1954, with the enzyme subsequently named thymidine phosphorylase, now commonly referred to as TYMP (1). Due to the technological limitations of the time, research on TYMP was limited following its discovery. It wasn’t until 1978 that Kubilus and Baden isolated and purified human TYMP from human amniotic membrane, confirming its existence in humans (2). This marked the beginning of a deeper exploration into the presence and functions of TYMP within the human body. Over time, TYMP’s essential biological roles, including promoting platelet activation, osteoclast differentiation, angiogenesis, and its involvement in tumor angiogenesis, epigenetic gene modification, apoptosis resistance, and nucleoside metabolic salvage, have been gradually unveiled. As such, TYMP has become a critical target in cancer research. Despite significant advancements, ongoing studies continue to deepen our understanding of TYMP’s full potential, yet much remains to be uncovered.

Cancer remains the leading cause of death worldwide (3–5). Its insidious nature means that by the time clinical symptoms become apparent, the disease is often in its advanced stages (6). While outcomes vary among patients, many advanced cancer cases can benefit from careful preoperative assessment and postoperative neoadjuvant chemotherapy, leading to improved prognoses (7, 8). However, these approaches do not diminish the significance of targeted cancer therapies in the treatment landscape. In recent years, the development of targeted cancer therapies, coupled with clinical trials, has provided promising alternative treatment options, offering patients greater choices. Therefore, understanding the underlying mechanisms of cancer and identifying viable therapeutic targets, such as TYMP, has become an essential focus in cancer research.

## 2 Structure of TYMP

### 2.1 Gene and molecular structure of TYMP

The TYMP gene is located on chromosome 22q13.32-qter (9). Whether in mammals or bacteria, TYMP exhibits structural conservation, existing as an anionic protein composed of two homodimers with a relative molecular mass of 55,000. Notably, when investigating the three-dimensional structure of *Escherichia coli* thymidine phosphorylase, researchers determined its active site by differentiating thymine and thymidine binding. This marked the first reported identification of a possible molecular structure of TYMP with thymine in the active site (10). Furthermore, human TYMP shares 39% sequence homology with *Escherichia coli* TYMP (11). Human TYMP possesses a proline-rich N-terminus (12), a feature absent in bacterial TYMP, potentially explaining the functional differences between the two. For instance, human TYMP plays a role in promoting platelet activation and hemostasis, functions that bacteria do not require.

### 2.2 TYMP enzyme activity and TYMP inhibitors

TYMP serves as a rate-limiting enzyme (13), exhibiting the common characteristic where enzyme structure influences its activity (14–16). Tipiracil hydrochloride (TPI) has been identified as a potent TYMP inhibitor, demonstrating high binding affinity to the enzyme. Moreover, TPI can function as an imaging agent for assessing TYMP expression *in vivo*, owing to its stability post-18 F labeling (17). Another notable TYMP inhibitor is the tritylated inosine derivative 5’-O-triynyl sugar (formerly KIN59), which is a non-competitive inhibitor (16). KIN59 binds to TYMP, inducing a conformational change that inhibits enzymatic activity without affecting substrate binding, underscoring the impact of TYMP conformational alterations on its enzymatic function. Additionally, Karen et al. identified polycyclic nitrogen heterocycles as potential TYMP inhibitors (18). Utilizing molecular docking techniques, their research demonstrated the interaction between polycyclic nitrogen heterocycles and TYMP’s active binding site. These findings highlight TYMP inhibitors as a promising class of drugs with significant research potential. For the development of TYMP-targeted therapies in the medical field, understanding the binding site and conformational changes of TYMP will be crucial for optimizing drug efficacy.

## 3 Physiological functions of TYMP

### 3.1 Downstream activation pathways of TYMP

**Figure 1:**
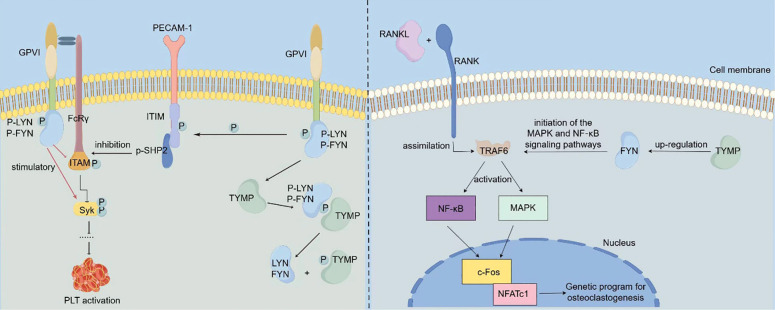
Mechanisms via which TYMP promotes platelet activation and osteoclast differentiation [The left-hand panel was adapted from Li et al. (2014) (12)]. LYN and FYN bind to the cytoplasmic domain of one GPVI molecule, while the other GPVI molecule interacts with the FcRγ chain dimer, thereby initiating the GPVI signaling pathway. Activation of this pathway results in LYN and FYN stimulating the phosphorylation of ITAM and Syk, which in turn activates platelet activation. Concurrently, LYN inhibits collagen-induced platelet activation by promoting the phosphorylation of the immunoreceptor tyrosine inhibitory motif (ITIM) domain of platelet endothelial cell adhesion molecule 1 (PECAM1). TYMP binds to the phosphate group of p-LYN, dephosphorylating it and causing the loss of its ability to mediate PECAM-1/ITIM phosphorylation. This action attenuates LYN's inhibitory function on collagen-induced platelet activation, thereby indirectly promoting platelet activation and contributing to thrombosis. In OC precursor cells, RANKL binds to the RANK receptor on OC precursors, leading to the recruitment of TRAF6, which activates NF-kB and AP-1 transcription factors, triggering downstream signaling pathways. This activation promotes the expression of c-Fos and NFATc1, key drivers of osteoclast differentiation. In TYMP-stimulated cells, the expression of FYN is significantly increased. TYMP activates FYN signaling, which subsequently promotes the activation of MAPK and NF-kB signaling pathways, thereby facilitating osteoclast differentiation.

TYMP performs a variety of functions, with its activities often relying on multiple signal transduction pathways. In recent years, research into TYMP has advanced significantly, leading to a deeper understanding of its mechanisms. Among the key mechanisms of TYMP are the following:

Platelets, which play a pivotal role in thrombosis, rely on platelet activation as a critical factor in the thrombotic process. Collagen-induced platelet activation is primarily mediated through glycoprotein VI (GPVI). GPVI exists in both monomeric and dimeric forms on the cell surface and is associated with various effectors, including the Fc receptor γ chain (FcRγ), spleen tyrosine kinase, phospholipase Cγ, protein kinase Cδ, and bisphosphoinositide polyphosphate phosphodiesterase 1 (19). During platelet activation, LYN and FYN kinases bind to the cytoplasmic domain of one GPVI molecule, while the other GPVI molecule associates with the FcRγ chain dimer, thereby activating the GPVI signaling pathway that mediates platelet activation (20). Src family kinases (SFKs) play a pivotal role in this activation cascade (21), with FYN acting as the primary stimulator of SFKs, which in turn influence platelet activation. LYN can suppress collagen-induced platelet activation by promoting phosphorylation of the immunoreceptor tyrosine inhibitory motif (ITIM) domain of platelet endothelial cell adhesion molecule 1 (PECAM1) (22, 23). TYMP interacts with the phosphate group on p-LYN, removing it and impairing LYN’s ability to mediate PECAM-1/ITIM phosphorylation. This action reduces LYN’s inhibitory function on collagen-induced platelet activation, thereby indirectly promoting platelet activation and thrombosis (12). This mechanism of TYMP’s role in platelet activation is summarized in **Figure 1**. Consequently, targeting TYMP with specific drugs may offer a promising approach for developing new anti-platelet and anti-thrombotic treatments.

The receptor activator of nuclear factor-κB ligand (RANKL)-associated signaling pathway plays a critical role in osteoclastogenesis and is potentially linked to inflammation (24). Osteoclasts (OCs) are multinucleated hematopoietic cells capable of bone resorption. Their formation is supported by macrophage colony-stimulating factor (M-CSF) and RANKL, which binds to the RANK receptor on OC precursors (25). The RANK-RANKL interaction recruits tumor necrosis factor receptor-associated factor 6 (TRAF6), which activates the NF-κB and AP-1 transcription factors (26, 27), initiating the signaling cascade. This activation promotes the expression of key molecules such as c-Fos, Cathepsin K, and T cell nuclear factor cytoplasmic 1 (NFATc1), thereby facilitating OC differentiation (28, 29) (**Figure 1**). Once differentiated, OC cells are rapidly driven toward apoptosis. FYN plays a key role in stimulating osteoclastogenesis by enhancing the proliferation and differentiation of OC precursors (30). This effect is primarily mediated through phosphorylation. A Japanese research team identified TYMP as a factor that induces osteoclast differentiation by activating FYN signaling. After TYMP stimulation, they observed a marked increase in phosphorylated FYN levels and enhanced FYN gene expression in macrophages. Additionally, sedimentation experiments revealed that TYMP binds to a complex containing integrin β1 (ITGβ1) and FYN. Furthermore, in TYMP-stimulated cells, there was a significant upregulation of phosphorylated protein kinase 1/2 (MEK1/2) and phosphorylated extracellular signal-regulated kinase 1/2 (ERK1/2). These findings suggest that TYMP activates FYN signaling, leading to the activation of MAPK and NF-κB pathways, thereby promoting osteoclast differentiation (31). The detailed mechanism by which TYMP promotes osteoclast differentiation is summarized in **Figure 1**.

### 3.2 Pro-angiogenic function of TYMP

Angiogenesis, the process of forming new blood vessels from existing ones, is promoted by TYMP. TYMP is highly expressed not only in tumor cells but also in normal cells, including macrophages, stromal cells, glial cells, and certain epithelial cells (32). Notably, the elevated expression of TYMP in macrophages and skin plays a critical role in maintaining systemic thymidine homeostasis.

TYMP catalyzes the conversion of thymidine (TdR) into deoxyribose-1-phosphate (dR-1-P) and thymine, producing a molecular structure that contains deoxyribose (dR) (33). This dR component can further participate in angiogenesis (34). Since TYMP catalyzes this reaction and indirectly influences angiogenesis, its pro-angiogenic effect is closely tied to its enzymatic activity. Consequently, inhibiting TYMP’s enzymatic activity with TYMP inhibitors to block its pro-angiogenic effects presents a potential therapeutic strategy. Furthermore, TYMP can directly induce angiogenic factors such as interleukin-8, basic fibroblast growth factor, and tumor necrosis factor α, which promote angiogenesis and stimulate endothelial cell migration and invasion (33). However, research into TYMP’s role in angiogenesis remains limited, and it is unclear whether additional pro-angiogenic mechanisms exist. Therefore, the full extent of TYMP’s involvement in angiogenesis is not yet fully understood, and further investigation is necessary.

### 3.3 Genetic disorders caused by TYMP deficiency

Mitochondrial neurogastrointestinal encephalomyopathy (MNGIE) is a rare autosomal recessive disorder caused by mutations in the TYMP gene, resulting in the loss of TYMP function (35). This disease leads to dysfunction in the digestive and nervous systems, presenting with clinical symptoms such as cachexia, ptosis, external ophthalmoplegia, peripheral neuropathy, and leukoencephalopathy (36–38). While no significant side effects have been reported for TYMP inhibitors as emerging drugs, the existence of MNGIE serves as a reminder that TYMP inhibitors could have previously unrecognized side effects. Therefore, extensive research is still needed before TYMP inhibitors can be applied clinically.

## 4 Role of TYMP in the occurrence and progression of cancer

**Figure 2:**
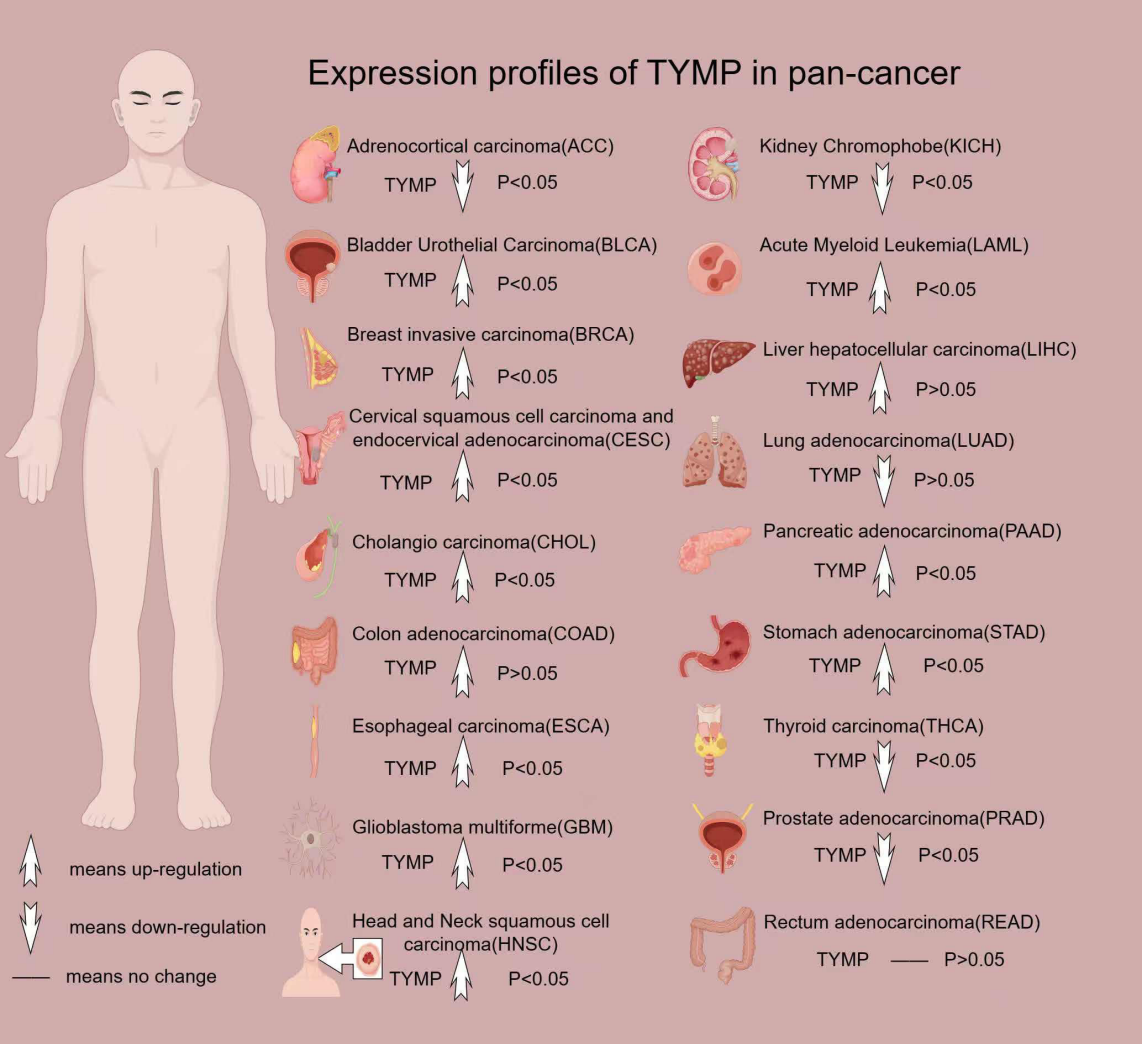
Expression profile of TYMP in pan-cancer. All data in this image are sourced from the GEPIA2 platform.

TYMP is overexpressed in various cancers, including breast cancer (38), gastric cancer (39), esophageal cancer (40), oral squamous cell carcinoma (41), lung cancer (42), colorectal cancer (43), cervical cancer (44), and bladder cancer (45). Furthermore, plasma levels of TYMP in individuals with certain cancers are significantly higher than in healthy individuals (46) (**Figure 2**).

### 4.1 TYMP-driven tumorigenesis through angiogenesis

The most well-established function of TYMP is its role in promoting angiogenesis. For tumors to proliferate extensively, they require a constant supply of oxygen and nutrients, which depend on blood circulation. Thus, a fully developed vascular system is essential for tumor growth and progression. TYMP’s angiogenesis-promoting effect addresses this need. As previously mentioned, TYMP facilitates angiogenesis through the dR component in its metabolites and by directly stimulating angiogenic factors, which in turn influence tumor growth and development. Consequently, inhibiting TYMP enzymatic activity using TYMP inhibitors has emerged as a viable therapeutic approach for slowing cancer progression. However, not all tumors depend on TYMP for angiogenesis. Some tumors, despite having dense microvascular networks, show no significant increase in TYMP levels (47). This suggests that additional, yet unexplored, mechanisms may also drive tumor angiogenesis. Nonetheless, it is clear that tumors with high TYMP expression are likely to have dense microvascular systems.

### 4.2 TYMP-driven tumorigenesis through epigenetic modification

**Figure 3:**
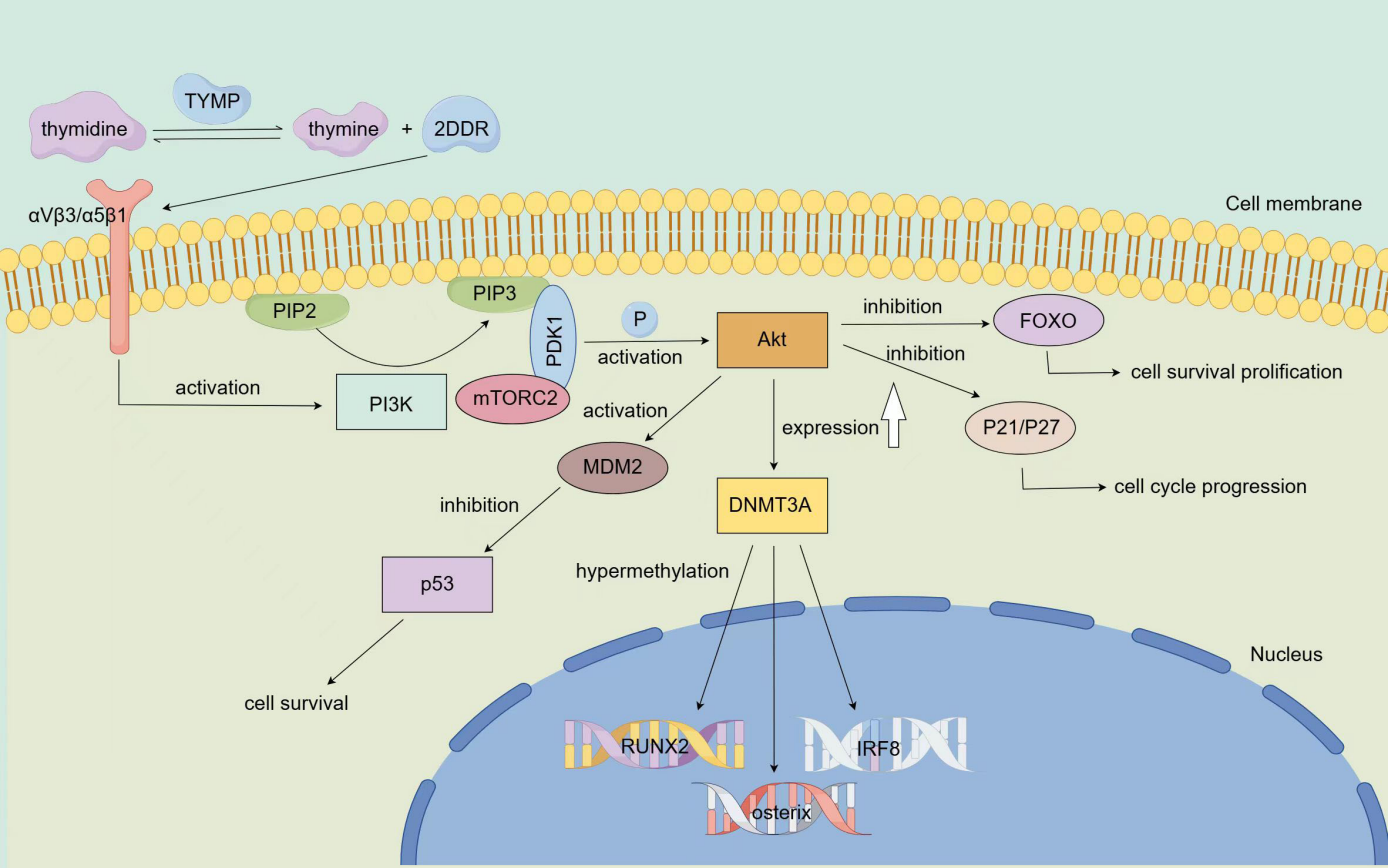
Mechanism *via* which TYMP modifies epigenetic and resists apoptosis TYMP catalyzes the conversion of thymidine to thymine and 2DDR, which binds to integrin αVβ3/α5β1 on progenitor cells, thereby activating the PI3K pathway. Once activated, PI3K promotes the conversion of PIP2 to PIP3, leading to the phosphorylation of Akt *via* PDK1 and mTORC2, completing Akt activation. Activated Akt inhibits FOXO, a key initiator of apoptosis, thereby promoting cell survival. Additionally, Akt can enhance tumor growth by suppressing the expression of tumor suppressor proteins P21 and P27. Furthermore, Akt acts on the MDM2 oncoprotein, negatively regulating the p53 tumor suppressor, thus resisting apoptosis and promoting tumor cell survival. Activated Akt also upregulates the expression of DNMT3A, leading to the hypermethylation of key genes such as RUNX2, osterix, and IRF8.

TYMP also influences tumor progression through its role in DNA methylation regulation. A 2016 study revealed that TYMP catalyzes the conversion of thymidine to thymine and 2-deoxy-D-ribose (2DDR), which then binds to integrin αVβ3/α5β1 on progenitor cells, activating the PI3K/Akt signaling pathway. This results in increased expression of DNA methyltransferase 3A (DNMT3A), leading to hypermethylation of key genes such as RUNX2, osterix, and IRF8 (48) (**Figure 3**). This mechanism is particularly significant in myeloma, where TYMP-induced hypermethylation of these genes contributes to reduced bone formation and enhanced bone resorption. TYMP overexpression is commonly observed in bone metastatic tumors, further supporting the potential of TYMP-targeted therapies for treating myeloma-related bone diseases.

### 4.3 TYMP-driven tumorigenesis through anti-apoptotic pathways

TYMP plays a key role in helping tumor cells resist apoptosis induced by hypoxia. Kitazono’s study demonstrated that TYMP’s metabolites, 2-deoxy-D-ribose and thymine, can partially counteract hypoxia-induced apoptosis in KB/TP cells by transfecting them with endothelial cell growth factor/thymidine phosphorylase (PD-ECGF/TP) cDNA (49). Additionally, research involving myocardial ischemia in dogs showed that PD-ECGF/TP improved ischemic conditions, alleviating apoptosis caused by hypoxia (50, 51). Furthermore, TYMP activates the PI3K/Akt signaling pathway (48), a key pathway involved in regulating cell apoptosis (52). Upon PI3K activation, phosphatidylinositol-4,5-bisphosphate (PIP2) is converted into phosphatidylinositol-3,4,5-triphosphate (PIP3) (53), leading to the phosphorylation of Akt *via* phosphoinositide-dependent kinase 1 (PDK1) and mammalian target of rapamycin complex 2 (mTORC2), thereby activating Akt (54). Activated Akt inhibits the Forkhead box O (FOXO) transcription factors, which initiate apoptosis, thereby promoting cell survival (55). Additionally, Akt can inhibit the expression of cell cycle regulators P21 and P27, further supporting tumor growth (56). Akt also negatively regulates the p53 tumor suppressor protein *via* MDM2, resisting apoptosis and promoting tumor cell survival (57) (**Figure 3**). Thus, TYMP can prevent cell apoptosis by activating the PI3K/Akt pathway, supporting tumor cells in resisting apoptosis induced by various treatments such as the immune response, hypoxia, radiotherapy, and chemotherapy. Understanding TYMP’s anti-apoptotic role could enhance cancer therapies and offer insights into treating diseases caused by cellular hypoxia. The main signaling pathways of TYMP are summarized in **Figure 3**.

### 4.4 TYMP-driven tumorigenesis through stabilizing the thymidine pool

As a key enzyme in the nucleoside metabolic salvage pathway, TYMP helps maintain thymine pool stability, a key factor for DNA synthesis. Tumor cells reprogram their metabolic pathways to meet growth demands, with pyrimidine metabolism playing a central role. Studies have shown that the expression levels of thymidylate synthase (TYMS) and TYMP in tumor tissues are significantly higher than in adjacent normal tissues. Both TYMS and TYMP mRNA have the potential to serve as reliable diagnostic indicators for colon cancer (CC), but further research is required to confirm this (58). Given that tumor growth depends on cell division, TYMP’s involvement in cell proliferation and tumor development suggests its critical role in cancer progression. Further investigation into the relationship between TYMP and tumors is needed.

## 5 Roles of TYMP in anti-cancer therapy

### 5.1 TYMP functions as a target for cancer therapy

Although TYMP exerts several promoting effects on tumor cell growth and development, it also holds therapeutic potential in cancer treatment. As a molecular target, TYMP is being explored for the development of cancer therapeutics (59). Investigating its biological functions in tumors may lead to the synthesis of TYMP inhibitors, which could prevent angiogenesis and slow tumor metastasis. However, research has indicated that certain TYMP activities can activate a variety of chemotherapeutic agents (40), which is crucial for cancer therapy. For instance, bevacizumab can enhance the metabolic activation of 5-fluorouracil (5-FU) through upregulation of TYMP (60). Furthermore, recent studies suggest that utilizing human mesenchymal stem cells (hMSCs) as carriers to deliver TYMP to cancer cells may facilitate the conversion of docifluridine (5′-DFUR) into the toxic 5-FU, promoting cancer cell death (61).

The activation of nucleic acid sensors in endothelial cells (ECs) triggers inflammation in various diseases, including cancer. Specifically, activation of the cytoplasmic RNA sensor retinoic acid-induced gene 1 (RIG-I) significantly contributes to decreased EC survival, with TYMP being the most upregulated gene in this process. Therefore, inhibiting TYMP could potentially alleviate endothelial dysfunction and enhance cancer treatment (62).

As previously discussed, TYMP activity can activate chemotherapy drugs, while TYMP inhibitors could mitigate its angiogenic effects, thereby indirectly impeding tumor cell growth. We summarized the TYMP inhibitors that are currently of certain research value and the chemotherapeutic drugs that rely on TYMP activity activation (**Table 1**). Although TYMP inhibitors may hold promise in cancer therapy (69), their safety remains uncertain, and long-term research is necessary to determine their clinical viability.

**Table 1:** TYMP targeted drugs and their introduction.

Medicine	Introduction	References
2-thioxo-pyrazolo[1,5-a] [1,3,5]triazin-4-ones	A series of 2-thioxo-pyrazolo[1,5-a] [1,3,5]triazin-4-one derivatives were designed and synthesized, and their TYMP inhibitory potential was evaluated. Certain compounds were found to exhibit promising TYMP inhibitory activity. This provides a new direction for the design of novel TYMP inhibitors.	(63)
Ciprofloxacin analogs	A research team synthesized a series of ciprofloxacin and evaluated their inhibitory potential against TYMP. They found that some of the analogs had good inhibitory activity against thymidine phosphorylase. This drug may provide a new approach for treating tumors.	(64)
Tritylated inosine derivative 5'-O-triynyl sugar (formerly known as KIN59)	Acts as a non-competitive inhibitor of TYMP.	(65)
Polycyclic nitrogen heterocycles	As a potential TYMP inhibitor, it has certain research value.	(18)
Tipiracil hydrochloride (TPI)	TPI is a selective TYMP inhibitor that exerts its antithrombotic effect by blocking TYMP-mediated platelet activation through the inhibition of TYMP-LYN binding. The safety profile of TPI has been confirmed in experimental studies, demonstrating a lower bleeding risk even at high doses compared to commonly used clinical agents such as aspirin and clopidogrel. Currently, TPI has been approved for clinical use by the U.S. Food and Drug Administration (FDA).	(66)
Docifluridine and TYMP-expressing mesenchymal stem cells	Human mesenchymal stem cells are used as delivery vehicles to deliver a certain amount of TYMP activity to docifluridine, a prodrug of 5-fluorouracil, thereby converting the non-toxic prodrug docifluridine into the toxic chemotherapy drug 5-fluorouracil, thereby eliminating cancer cells.	(67)
Bevacizumab	Bevacizumab mediates the activation of 5-fluorouracil by upregulating TYMP, thereby achieving a therapeutic effect on tumors.	(60)
Capecitabine	Capecitabine relies on TYMP to activate the intermediate form 5'-deoxy-5-fluorouracil into the active form 5-fluorouracil, thereby achieving a therapeutic effect on tumors.	(68)

### 5.2 Targeting TYMP to alleviate resistance to cancer immunotherapy and chemotherapy

Immunotherapy and chemotherapy are widely used in cancer treatment; however, their efficacy is not always consistent. Over prolonged exposure to these therapies, tumor cells often develop drug resistance, which diminishes treatment effectiveness. This phenomenon is a key factor contributing to the continued challenges in achieving significant progress in cancer treatment.

Peri et al. observed the upregulation of TYMP in gastric cancer cells that were induced to become resistant to 5-fluorouracil, identifying TYMP gene mutations in tumor cells as a common cause of 5-fluorouracil resistance. Such mutations can enhance angiogenesis in gastric cancer cell lines (70). Another study found that TYMP-induced T cell exhaustion plays a critical role in immunotherapy resistance in colorectal cancer (CRC) (71). Targeted TYMP therapies could potentially improve the effectiveness of immunotherapy. In 2022, a research team explored whether demethylation of TYMP would increase cancer cell sensitivity to 5-FU. While the results did not demonstrate that TYMP demethylation alone enhanced 5-FU sensitivity, they suggested a potential strategy combining TYMP with other metabolic pathways to boost 5-FU responsiveness (72). These findings provide novel insights into drug resistance in tumor cells, thereby enhancing cancer treatment strategies.

### 5.3 Role of TYMP in prognostic evaluation of tumor therapies

The expression of TYMP is closely associated with rectal cancer treatment outcomes (73). Preoperative radiotherapy or chemoradiotherapy (CRT) is considered the standard treatment for locally advanced rectal cancer (74, 75), and studies have shown that TYMP expression can help predict the efficacy of CRT (76). Furthermore, TYMP’s significance in CRC progression is evident, as a study demonstrated that the rs11479 polymorphism could predict the prognosis of patients with CRC receiving capecitabine-based adjuvant chemotherapy (77). This highlights that modulating TYMP mRNA expression could enhance the effectiveness of capecitabine-based therapy, thus improving survival rates in patients with CRC.

Additionally, TYMP expression in CRC tumor epithelial cells is linked to recurrence-free survival (RFS) in patients with CRC. Elevated TYMP expression may correlate with poor prognosis in these patients (78). Furthermore, research has shown that TYMP polymorphisms can influence the prognosis of patients with CRC undergoing chemotherapy by modulating TYMP mRNA expression (79, 80).

TYMP also serves as a valuable immune prognostic marker in various cancers, including renal clear cell carcinoma (81, 82) and low-grade glioma (83). Recent studies have shown that a reduction in TYMP expression can significantly affect the proliferation, migration, and invasion of renal cell carcinoma (RCC) cells *in vitro (* 84). Furthermore, a 2016 study indicated a potential link between TYMP levels and the survival rate of patients with localized gastric cancer following radical gastrectomy (85). Additionally, the combined expression of TYMP and hypoxia-inducible factor α (HIF-1α) has been shown to predict the prognosis of patients with rectal cancer undergoing neoadjuvant chemoradiotherapy with oxaliplatin and capecitabine (XELOXART) (86). These findings underscore the pivotal role of TYMP in cancer treatment and prognosis prediction.

Despite its promising potential in cancer therapy, TYMP has certain limitations. Due to individual differences among patients, TYMP-targeting drugs may be effective for some cancer individuals, while less effective for others. Moreover, TYMP not only promotes tumor progression but also influences normal physiological functions, making it crucial to consider the potential side effects of TYMP-targeted therapies. Further research and clinical trials are needed to validate these effects. While significant progress has been made in understanding TYMP’s role in disease, many aspects remain to be explored. The mechanisms underlying TYMP’s involvement in tumors are complex, and the clinical effects of inhibiting TYMP have yet to be fully established. The potential for unknown side effects of TYMP inhibitors also warrants further investigation. Despite these uncertainties, TYMP remains a promising therapeutic target. It holds considerable potential in the understanding and treatment of cancer, making ongoing research into TYMP essential. However, considering the current state of research, significant progress is still required before TYMP-targeted therapies can be fully developed and applied.

## 6 Conclusions

TYMP, a protein found extensively in both prokaryotic and eukaryotic cells, performs critical physiological functions, including promoting platelet activation, osteoclast differentiation, and angiogenesis. Mutations in the TYMP gene can lead to genetic disorders such as MNGIE. Beyond its role in normal cells, TYMP is also pivotal in cancer cells, where it facilitates tumor angiogenesis, modifies epigenetics, and helps resist cell apoptosis. Additionally, TYMP functions as a key enzyme in the nucleoside metabolic rescue pathway. Consequently, studying TYMP enhances our understanding of cancer mechanisms and is vital for cancer treatment. Targeting TYMP has emerged as a promising strategy for cancer therapy, and TYMP-activated anti-tumor drugs represent an important therapeutic approach. Despite existing limitations, further research on TYMP holds significant potential for advancing cancer treatment.
