# Correction: Thymidine phosphorylase in nucleotide metabolism: physiological functions and its implications in tumorigenesis and anti-cancer therapy

**DOI:** 10.3389/fimmu.2025.1642752

**Published:** 2025-07-09

**Authors:** Bo Huang, Qihang Yuan, Jiaao Sun, Chao Wang, Dong Yang

**Affiliations:** ^1^ Liaoning Cancer Hospital & Institute, Shenyang, China; ^2^ First Affiliated Hospital of Dalian Medical University, Dalian, China

**Keywords:** nucleotide metabolism, thymidine phosphorylase, physiological functions, tumorigenesis, anticancer therapy

In the published article, there was an error in the legend for **Figure 1** Mechanisms via which TYMP promotes platelet activation and osteoclast differentiation. as published. The left-hand part of **Figure 1**, illustrating the mechanism by which TYMP regulates platelet activation, was adapted from Figure 7 of the paper by Li et al. (2014), titled “Thymidine Phosphorylase Participates in Platelet Signaling and Promotes Thrombosis” (https://doi.org/10.1161/CIRCRESAHA.115.304591). However, we did not indicate this in the figure legend. The corrected legend appears below.

**Figure 1:**
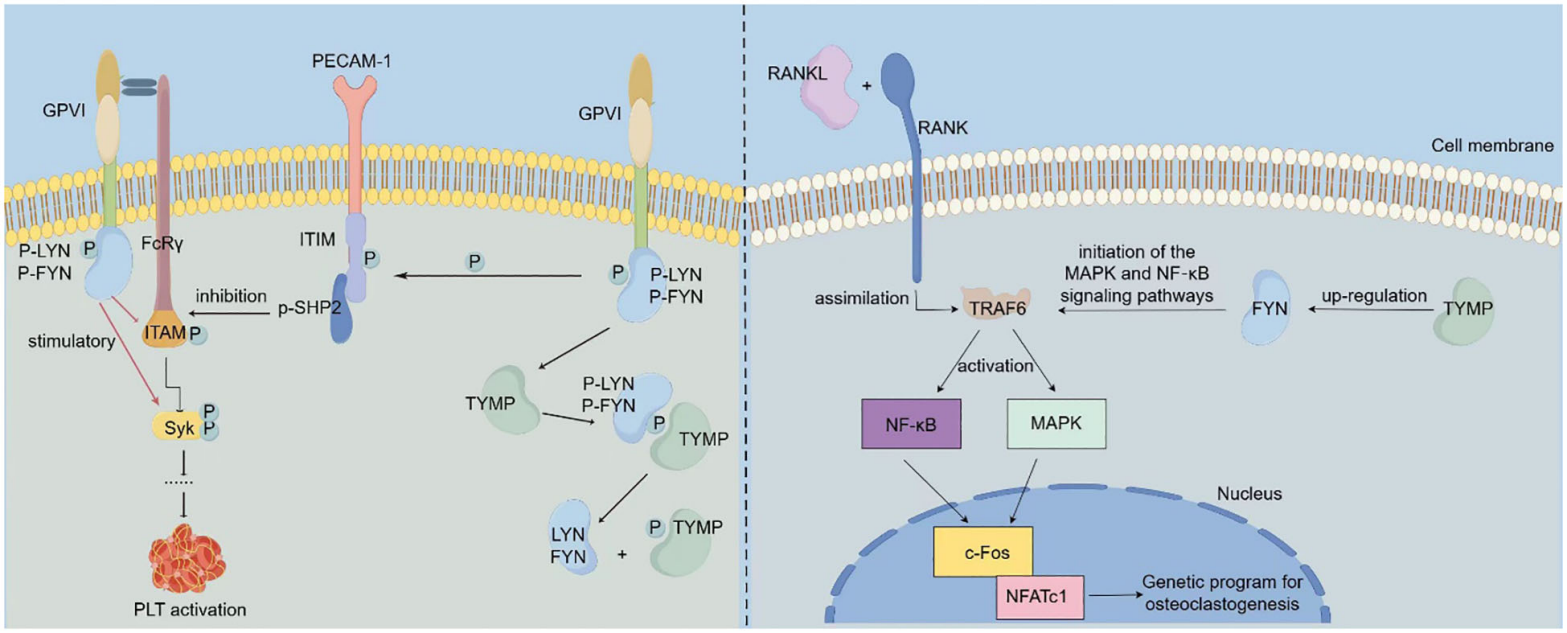
Mechanisms via which TYMP promotes platelet activation and osteoclast differentiation (The left-hand panel was adapted from Li et al. (2014) (12)). LYN and FYN bind to the cytoplasmic domain of one GPVI molecule, while the other GPVI molecule interacts with the FcR**γ** chain dimer, thereby initiating the GPVI signaling pathway. Activation of this pathway results in LYN and FYN stimulating the phosphorylation of ITAM and Syk, which in turn activates platelet activation. Concurrently, LYN inhibits collagen-induced platelet activation by promoting the phosphorylation of the immunoreceptor tyrosine inhibitory motif (ITIM) domain of platelet endothelial cell adhesion molecule 1 (PECAM1). TYMP binds to the phosphate group of p-LYN, dephosphorylating it and causing the loss of its ability to mediate PECAM-1/ITIM phosphorylation. This action attenuates LYN’s inhibitory function on collagen-induced platelet activation, thereby indirectly promoting platelet activation and contributing to thrombosis. In OC precursor cells, RANKL binds to the RANK receptor on OC precursors, leading to the recruitment of TRAF6, which activates NF-kB and AP-1 transcription factors, triggering downstream signaling pathways. This activation promotes the expression of c-Fos and NFATc1, key drivers of osteoclast differentiation. In TYMP-stimulated cells, the expression of FYN is significantly increased. TYMP activates FYN signaling, which subsequently promotes the activation of MAPK and NF-kB signaling pathways, thereby facilitating osteoclast differentiation.

The original version of this article has been updated.

